# Identification of Potential Plant-Derived Pancreatic Beta-Cell-Directed Agents Using New Custom-Designed Screening Method: *Gymnema sylvestre* as an Example

**DOI:** 10.3390/molecules29010194

**Published:** 2023-12-28

**Authors:** Altaf Al-Romaiyan, Shanta J. Persaud, Peter M. Jones

**Affiliations:** 1 Department of Pharmacology and Therapeutics, College of Pharmacy, Kuwait University, Jabriya 046302, Kuwait; 2 Department of Diabetes, School of Cardiovascular Medicine & Sciences, Faculty of Life Sciences and Medicine, King’s College London, London SE1 1UL, UK; shanta.persuad@kcl.ac.uk (S.J.P.); peter.jones@kcl.ac.uk (P.M.J.)

**Keywords:** diabetes, screening, plant-derived therapies, beta-cell function, insulin, apoptosis

## Abstract

Background: Folk medicines are attractive therapeutic agents for treating type 2 diabetes mellitus (T2DM). Most plant extracts that have been suggested to restore β-cells function were tested in vivo. Some only have been tested in vitro to determine whether they have a direct effect on β-cells islets of Langerhans. Currently, there are no defined criteria for screening of β-cell-directed plant-based remedies as potential antidiabetic agents. Summary: In this review, we have identified certain criteria/characteristics that can be used to generate a “screening portfolio” to identify plant extracts as potential β-cell-directed agents for the treatment of T2DM. To validate our screening method, we studied the potential therapeutic efficacy of a *Gymnema sylvestre* (GS) extract using the screening criteria detailed in the review. Six criteria have been identified and validated using OSA^®^, a GS extract. By using this screening method, we show that OSA^®^ fulfilled most of the criteria identified for an effective β-cell-directed antidiabetic therapy, being an effective insulin-releasing agent at nontoxic concentrations; maintaining β-cell insulin content by stimulating a concomitant increase in insulin gene transcription; maintaining β-cell mass by protecting against apoptosis; and being effective at maintaining normoglycemia in vivo in a mouse model and a human cohort with T2DM. Key messages: The present review has highlighted the importance of having a screening portfolio for plant extracts that have potential antidiabetic effects in the treatment of T2DM. We propose that this screening method should be adopted for future studies to identify new β-cell-directed antidiabetic plant derived agents.

## 1. Introduction

The area of alternative and complementary medicine, herbal and plant-based remedies in particular, has been the focus of attention of scientists in the past few decades, and it has been estimated that the percentage of people who have used herbal medicine reached 35–48% worldwide [1,2]. Diabetes mellitus is a health problem affecting all populations globally. The prevalence of the disease is increasing, and by the year 2045, the estimated number of adult people who will develop diabetes will rise to 629 million [3]. Type 2 diabetes mellitus (T2DM), which is characterized by two important pathophysiological characteristics, namely β-cell dysfunction and impaired insulin responsiveness in insulin target tissues, will affect 95% of all diabetes cases.

It is clear that β-cell dysfunction is the cornerstone in the development of T2DM and symptoms of hyperglycemia appear when β-cell function is no longer sufficient to maintain glucose homeostasis. Several studies have reported that the primary trigger for overt T2DM is impaired β-cell function and that reduced β-cell function/loss of β-cell mass is a determinant of the severity of T2DM [4,5,6]. Furthermore, variations in several genetic loci have been reported, and these loci are associated with an increased risk of developing T2DM [7,8,9,10,11,12]. The involvement of most genes in controlling β-cell function and insulin secretion supports the role of β-cell dysfunction in the progression of T2DM. Therefore, enhancement of β-cell function is one of the major drug targets for the reduction in hyperglycemia and prevention of microvascular and macrovascular complications. Examples of currently available therapies that target β-cell function are sulphonylureas and glucagon-like peptide 1 (GLP-1)-based therapy. However, serious adverse effects are associated with some of these therapies, and complete glycemic control is difficult to achieve. Therefore, introducing and developing new drugs that target β-cell function, especially those of plant origin, continues to be an active area of research. Metformin, which was originally isolated from a plant named *Galega officinalis*, is one of the most widely used frontline antidiabetic drugs in the management of T2DM [13]. Thus, identifying novel, effective extracts of plant origin to modulate β-cell function and thus treat T2DM could offer more affordable and easily accessible antidiabetic alternatives in the developing world.

Many plant species have been claimed to be effective in treating T2DM [14]. However, there are currently no defined criteria for identifying and screening herbal remedies that have positive activities on β-cell function as potential antidiabetic agents. Therefore, in this review, we have identified a number of characteristics/properties that can be used as screening tools to identify and investigate plant extracts with potential effects on β-cells as potential therapeutic agents for the treatment of T2DM.

## 2. Screening Plant Extracts for Therapeutic Potential

**Figure 1:**
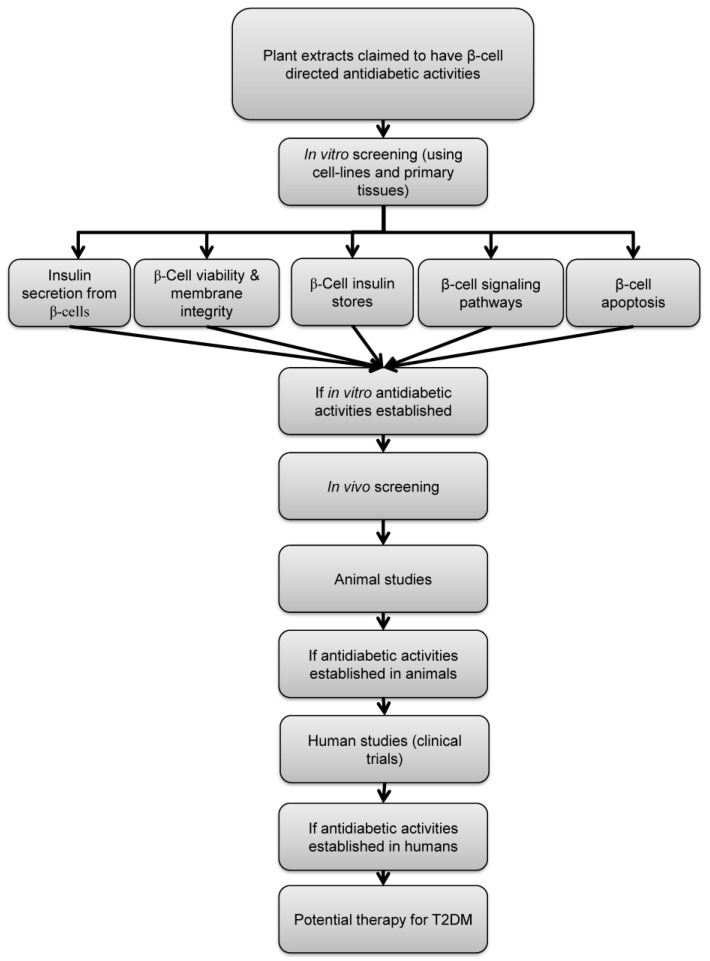
Schematic representation of the proposed screening criteria of plant-derived antidiabetic agents. Plants claimed to have antidiabetic activities by acting through modulation of β-cell function should be first screened in vitro for the following properties: stimulation of insulin secretion, maintaining β-cell viability, preservation of β-cell insulin store, activation of known β-cell signaling pathways and preservation of β-cell mass. If antidiabetic effect of a certain plant extract is established, confirmation of efficacy in animal models of diabetes and human subjects is required.

Here, we propose specific criteria that can be applied as a “screening portfolio” to identify potential β-cell-focused therapeutic agents of plant origin (Figure 1). Each plant extract will have an individualized profile that can act as a fingerprint for that extract, allowing rapid evaluation, comparison between extracts, and future identification of the most promising extracts that have the potential to be progressed to clinical trials and market.

A very promising plant extract should possess some or all of the following criteria/properties, with the most promising extracts possessing more of the properties, thus enabling the ranking of extracts for therapeutic potential:

1. Potentiating nutrient-induced insulin secretion to reduce postprandial hyperglycemia [15,16,17,18,19];
2. Stimulating insulin secretion while maintaining β-cell viability and preserving β-cell membrane integrity [17,18,19,20];
3. Stimulating insulin secretion while preserving β-cell insulin stores [15];
4. Activating identifiable steps in the β-cell stimulus–secretion coupling pathway [17,19,21];
5. Preserving β-cell mass by protecting against apoptosis [19,20];
6. Efficacy in animal models of diabetes and in human subjects in vivo [15,16,22,23,24,25,26,27,28,29,30,31,32,33,34,35,36,37,38,39].

We have applied these criteria to a plant that has been widely used in herbal medicine to treat the symptoms of T2DM, *Gymnema sylvestre.*

## 3. Methodology

In this review, the authors aimed at identifying potential β-cell-directed plant extract by recognizing some characteristics as a “screening portfolio” using *Gymnema sylvestre* as an example. A literature review was carried out. Central PubMed was searched to retrieve relevant scientific publications, using *Gymnema sylvestre* or its extract OSA^®^ as the main searched keyword combined with one or more of the following keywords: diabetes, β-cells, MIN6 cells, human study, animal model of diabetes, insulin secretion, cell viability, apoptosis, signaling pathway, mouse islets, human islets. Research articles published from inception until January 2023 were selected for data collection. Table 1 summarizes the effects of OSA^®^ and other GS extracts in vitro and in vivo.

## 4. *Gymnema sylvestre* as an Antidiabetic Agent

Gymnema is a genus in the Asclepiadaceae family. A family that normally contains 100 species, among which the species *Gymnema sylvestre* (GS) has been repeatedly reported to be an effective ayurvedic medicine in treating various diseases, particularly T2DM [40,41]. The reported antidiabetic effect of GS has been shown to be linked to extracts of GS leaves. To validate our screening method, we studied the potential therapeutic efficacy of a GS extract named OSA^®^ (Santal Solution, India) [42,43] using the criteria described above. OSA^®^ is a high molecular weight GS extract (>3000 Da), and it is prepared by extracting fresh GS leaves with aqueous alcohol according to protocols described in the US Patents 6,949,261 and 6,946,151 [42,43].

### 4.1. Direct Stimulation of Insulin Release from β-Cell Line and Primary Islets

The first obvious target of an antidiabetic plant extract is to directly stimulate insulin secretion from β-cell in the islets of Langerhans in an analogous manner to currently used therapeutic agents, such as sulphonylureas, meglitinides, and glucagon-like peptide-1 (GLP-1) analogs [44,45]. In vitro experiments using β-cell lines and isolated primary islets are required to establish and validate this effect. Numerous β-cell lines have been used in insulin secretion experiments for decades, including RIN, BRIN-BD11 and MIN6 cells. Each cell line has experimental advantages and disadvantages, which have been reviewed in detail elsewhere [46], but the MIN6 cell line offers a useful model, with relatively high insulin content and glucose responsiveness, to screen for effective insulinotropic agents. MIN6 cells can be grown and used as adherent monolayers or configured as three-dimensional islet like structures (*pseudoislets*, (PIs)) to more closely mimic primary islets [47].

Two types of secretion experiments can be performed, depending on the level of information required, these being static incubations and perifusions. Static incubations measure the accumulation of insulin secretion from a fixed number of cells over a single given time period (usually 30 or 60 min), whereas perifusion experiments measure insulin secretion in sequential samples collected from perifused cells or PIs at a given frequency (usually every one or two minutes for up to 2 h). Perifusions are much more labor-intensive, but they have the advantage of detecting changes in the rate, reversibility and duration of the insulin output over a minute-to-minute time scale. The optimum results are obtained when a plant extract reversibly and sustainably stimulates insulin secretion under hyperglycemic conditions with no effect on insulin output at basal (<5 mM) glucose levels, thus avoiding potential hypoglycemic side-effects, which are common with the currently used sulphonylurea class of antidiabetic drugs. Most of our static and perifusion secretion experiments use a definite substimulatory (basal, 2 mM) or supramaximal stimulatory (20 mM) concentration of glucose to clearly identify those agents which stimulate insulin release under postabsorptive conditions and are thus potentially hypoglycemic and those which only enhance glucose-induced insulin secretion and are thus therapeutically more desirable.

In static incubations using MIN6 monolayers and at a substimulatory glucose concentration, we found OSA^®^ to cause dose-dependent increases in insulin secretion [17]. Other GS extracts have also been reported to stimulate insulin secretion from MIN6 cells and other β-cell lines [18,19].

The kinetic profile of the OSA^®^ insulin secretory responses was evaluated in perifusion experiments with MIN6 PIs. When MIN6 PIs were perifused with substimulatory glucose solution, a sustained increase in insulin secretion was induced by OSA^®^, and this increase was reversible upon withdrawal of OSA^®^. The sustainability and maintenance of insulin secretion following OSA^®^ exposure may provide therapeutic benefits in long-term glycemic control and thus reduce dosing frequency, although careful dosage would be needed to avoid potential hypoglycemia.

Although β-cell lines provide a more readily accessible alternative to primary islets, all β-cell lines are derived from transformed insulinoma cells, which may differ phenotypically from normal cells, so the insulinotropic activities of any plant extracts should also be tested in primary tissues. The insulin-releasing effects of OSA^®^ were also seen in primary mouse and human islets, demonstrating a direct activity on primary β-cells. In mouse and human islets, OSA^®^ caused a similar effect to that seen in MIN6 cells. OSA^®^ stimulated insulin secretion, which was again maintained in the presence of OSA^®^ [15,16]. However, a major risk of increasing insulin secretion at substimulatory glucose concentrations could be the development of hypoglycemic episodes. Despite this risk, it is well known that sulphonylureas and meglitinides are still being used successfully in the treatment of T2DM despite the fact that both stimulate insulin release at basal glucose concentrations [48]. It indicates that OSA^®^ may be at least as effective as these commonly used antidiabetic drug classes.

In primary mouse and human islets, OSA^®^ also augmented insulin secretion induced by glucose in addition to its ability to initiate insulin secretion at substimulatory glucose concentrations [15,16]. At 20 mM glucose, a supramaximal stimulatory glucose concentration, OSA^®^ stimulated insulin release. The ability of OSA^®^ to increase insulin secretion over 20 mM glucose suggests that OSA^®^ may work irrespective of nutrient metabolism and, therefore, may stimulate insulin secretion by bypassing glucose metabolism and thus being effective in glucose unresponsive β-cells. This action of OSA^®^ is similar to exendin-4, a GLP-1 agonist. However, unlike exendin-4, OSA^®^ has the advantage of not being broken down in the GI tract and is thus bioavailable after oral administration. Other GS extracts were also reported to stimulate insulin secretion from isolated rat islets incubated in the presence of 2, 10 and 20 mM glucose, in agreement with results obtained from OSA^®^ [18].

### 4.2. Maintenance of Cell Viability

Some plant extracts have been reported to have a deleterious effect on cell viability because of disruptions in plasma membrane integrity caused by some of the active constituents of the plant, increasing membrane pore formation and, thus, the plasma membrane permeability [18,49,50]. Maintaining the integrity of the plasma membrane is essential for regulated exocytosis of insulin and in allowing meaningful in vitro tests to be conducted, so it is important to screen extracts for deleterious effects on cell viability. The Trypan Blue exclusion test is a rapid and simple qualitative test used to measure the integrity of a cell’s plasma membrane and, hence, viability. Trypan blue dye (MW ≈ 1000 Da) can enter permeable cells and has the ability to stain nuclei. Therefore, under a light microscope, non-viable cells appear blue. In addition to the Trypan Blue exclusion test, other viability tests such as ATP viability test (in our lab, we used CellTiter-Glo^®^ Luminescent Cell Viability Assay, Promega, Madison, WI, USA) can quantify ATP content in metabolically active viable cells and thus can determine the number of viable cells in a fast and accurate fashion. The higher the ATP levels, the higher the number of viable cells.

OSA^®^, at the therapeutic concentrations used to induce insulin secretion, did not cause any noticeable damage to the plasma membrane, as shown by the Trypan Blue exclusion test and ATP viability test [17,20]. Other GS extracts have shown similar results [18,19]

### 4.3. Preservation of β-Cell Insulin Stores

The gradual loss of insulin secretory responses associated with the continued use of insulin-secreting drugs over extended periods of time can be avoided by maintaining and sustaining insulin reserves in β-cells after prolonged stimulation of insulin secretion [32]. Therefore, to sustain β-cell insulin stores, a successful antidiabetic therapy for T2DM should both promote insulin secretion and insulin biosynthesis. Sulphonylureas eventually failed as a first-line treatment for T2DM due to their inability to promote insulin gene production, which causes β-cell depletion and necessitates the use of insulin replacement therapy.

Measurement of (pre)proinsulin (PPI) mRNA or protein expression, using RT-PCR techniques and immunoassay measurements, respectively, can be used to establish the effects of plant extracts on the maintenance of β-cell stores of insulin. Increasing insulin gene transcription and PPI mRNA levels, or increased mRNA translation in response to the plant, will maintain total insulin content and thus preserve insulin stores within β-cells to prevent exhaustion in response to prolonged stimulation.

OSA^®^ fulfills the requirements of promoting both insulin secretion and insulin production. OSA^®^ significantly stimulated the genetic expression of PPI at the mRNA levels in chronically treated mouse islets. In addition, in spite of OSA^®^ causing prolonged insulin secretion stimulation, the total intraislet insulin content of mouse islets treated with OSA^®^ remained unchanged in comparison to vehicle-treated mouse islets [15]. The elevations in PPI mRNA levels and maintained intraislet insulin stores documented in OSA^®^-treated mouse islets suggest that OSA^®^ may be advantageous over some of the insulin secretagogue medications in use today, such as sulphonylureas.

### 4.4. Activation of Identifiable Steps in β-Cell Stimulus–Secretion Coupling Pathways

The stimulus–secretion coupling pathways in β-cells have been studied since the 1970s and are now fairly well understood. Briefly, glucose is phosphorylated within β-cells by the low affinity, high specificity glucokinase and metabolized largely by oxidative phosphorylation to generate ATP. The subsequent changes in the ATP/ADP ratio result in the closure of an inwardly rectifying K^+^ channel (K_ATP_) in the plasma membrane with the consequent depolarization of the cell and the opening of L-type voltage-gated Ca^2+^ channels (VGCC). This allows a rapid influx of extracellular Ca^2+^, which triggers the exocytosis of insulin-containing secretory granules, thus initiating the glucose-induced insulin secretory response [51]. Other second messenger systems, such as the adenylate cyclase/cyclic AMP (cAMP), phosphatidylinositol 3-kinases (PI3K)/phosphatidylinositol (3,4,5)-trisphosphate (PIP3), Ca^2+^/Calmodulin and the phospholipase C (PLC)/Inositol trisphosphate (IP3)/diacylglycerol (DAG) pathways, are also activated by glucose and other secretagogues to contribute to the insulin secretory response through the activation of specific classes of serine/threonine protein kinase enzymes.

Although it is not essential to understand the exact cellular mode of action of insulinotropic drugs, it is very useful when considering the therapeutic implications of their use. For example, sulphonylurea drugs were in use for many decades before the identification of the K_ATP_ channel as their major site of action in the β-cell, but this mode of action explains how sulphonylureas can stimulate insulin secretion from glucose-unresponsive β-cells and why sulphonylureas can induce hypoglycemia. Thus, measuring the effects of plant extracts on elements of these stimulus–secretion coupling pathways can offer insight into their cellular modes of action.

In our lab, changes in intracellular Ca^2+^ ([Ca^2+^]_i_) in single β-cells are routinely measured by Ca^2+^ microfluorimetry using Ca^2+^-sensitive fluorescent dyes such as Fura-2. This technique depends on ratiometric estimations of [Ca^2+^]_i_ in response to differences in excitation spectra following fluorophore binding. Elevations in [Ca^2+^]_i_ concentrations in Fura-2-loaded β-cells were associated with the insulin secretory responses to OSA^®^ as assessed by Ca^2+^ microfluorimetry, and these [Ca^2+^]_i_ increases caused by OSA^®^ were inhibited in the presence of ethylene glycol tetraacetic acid (EGTA) or nifedipine demonstrating that OSA^®^ increases cytosolic Ca^2+^ by enabling Ca^2+^ entry via L-type VGCC [17,21]. Other GS extracts have shown similar results [18].

The involvement of second messengers and/or their downstream protein kinases can be investigated by evaluating the effects of either pharmacological inhibitors or siRNA knockdown of specific cellular targets involved in the stimulus–secretion coupling processes on extract-induced insulin secretory responses. These approaches have been applied to OSA^®^, but the downstream elements of the signaling pathway of OSA^®^-induced insulin secretion are still not fully understood.

Opening K_ATP_ channels by using diazoxide did not block OSA^®^-induced insulin secretion, suggesting that it was independent of β-cell depolarization. However, OSA^®^-induced insulin secretion was inhibited, albeit only partially, by nifedipine, a VGCC blocker, suggesting a partial involvement of L-type VGCC, and thus Ca^2+^ influx in the secretory responses. Similarly, OSA^®^-induced insulin secretion was also partially inhibited by staurosporine, a non-selective serine/threonine protein kinase inhibitor, implicating that protein kinase activation was also involved in the OSA^®^-induced insulin secretion [21]. It is unknown which type of protein kinase is responsible for the stimulation of insulin secretion by OSA^®^. Protein kinase Cαβ (PKCαβ) and calcium-calmodulin kinase II (CamKII) have been reported to be involved in insulin secretion regulation from β-cells. However, both kinases were demonstrated not to be implicated in OSA^®^-induced insulin secretion by the use of selective kinase inhibitors.

Cyclic adenosine monophosphate (cAMP) has been shown to stimulate insulin secretion by sensitizing the secretory machinery to [Ca^2+^]_i_ without changing [Ca^2+^]_i_ and, therefore, may suggest its involvement in OSA^®^’s ability to partially increase insulin secretion independently of [Ca^2+^]_i_. However, because OSA^®^ surprisingly decreased [cAMP]_i_ levels in β-cells in conjunction with enhancing insulin secretion, the insulin secretion produced by OSA^®^ was separated from cAMP production [21].

### 4.5. Preserving β-Cell Mass

Reduction in β-cell mass is a hallmark of T2DM. It can be precipitated by β-cell death through caspase-dependent and -independent pathways leading to induction of apoptosis. Induction of apoptosis is triggered by a constant increase in cytokines, glucose and free fatty acids [52,53,54,55,56,57,58]. Preserving β-cell mass through compacting apoptosis may provide an important technique to prevent T2DM deterioration and progression. One successful example that could maintain β-cell mass is exendin-4, a GLP-1 agonist, which increased neogenesis and inhibited apoptosis induced by interleukin-1 beta (IL-1β) in β-cells in vitro, indicating that exendin-4 could have a protective role of in β-cells [59]. Two classes of antidiabetic drugs, namely exendin analogs or dipeptidyl peptidase IV (DPPIV) inhibitors, which inhibit the degradation and thus the metabolism of endogenous GLP-1, are now being used as a promising T2DM treatment because they have the ability to reduce hyperglycemia by concomitantly stimulating insulin secretion and maintaining β-cell mass [60].

There are many colorimetric, luminescence and fluorescence assays that are commercially available to measure apoptosis levels in cells or tissues. We have used a luminescent luciferase assay (Caspase Glo 3/7^®^ assay, Promega) to measure the activation of caspase-3/7 in β-cells as an indicator of apoptosis and have shown that OSA^®^ may offer similar protective effects to exendin-4. Thus, OSA^®^ significantly reduced caspase-3/7 levels that were induced by cytokine in MIN6 cells and in primary mouse islets. Consistent with this protective effect against β-cell loss via apoptosis, OSA^®^ also reduced the expression of pro-apoptotic effectors and enhanced the expression of key antiapoptotic effectors [20]. The findings of these studies suggest that OSA^®^ may possess an additional potential mechanism of action as an antiapoptotic agent that may enable it to be an effective treatment for T2DM. Furthermore, the combination of OSA^®^’s cytoprotective properties and its insulin secretagogue function may allow its use as an alternative therapy in T2DM. Other GS extracts were also reported to reduce the levels of reactive oxygen species and oxidative stress in vitro [19].

### 4.6. Improvement of Glycemia In Vivo

If in vitro measurements of the properties described above suggest that a plant extract has therapeutic potential, it is then necessary to test its efficacy in lowering and/or maintaining blood glucose in vivo, first in animal models of T2DM and subsequently in human cohorts. Although it is very important at this stage to test the toxicity of plant extracts in vivo, experiments that are designed to examine these effects have been reviewed elsewhere [61]. In our study using ob/ob mice, a T2DM model characterized by hyperglycemia and profound obesity, a single oral dose of OSA^®^ (500 mg/kg) dramatically improved the glucose intolerance characteristic of these animals during an intraperitoneal glucose tolerance test [15]. Similarly, our studies in a small cohort of human subjects with T2DM produced promising results. Improvements in blood glucose levels pre- and postprandially were noticeable following 60 days of oral OSA^®^ (1 g/day) administration to patients with T2DM. Normalization of both fasting and postprandial blood glucose concentrations after OSA^®^ treatment was observed, with elevations in C-peptide and plasma insulin levels indicating that such improvement in glycemic control induced by OSA^®^ was because of a direct effect of OSA^®^ on β-cells [16]. The use of OSA^®^ as a β-cell-directed and insulin-releasing therapy for T2DM is strongly supported by these findings. The OSA^®^ benefits shown in both our animal and human investigations were consistent with other GS extracts’ known antihyperglycemic properties [22,23,24,25,26,27,28,29,30,31,32,33,34,35,36,37,38,39].

## 5. Conclusions and Future Directions

The work described in this review highlights the potential importance of plant-based β-cell-targeted remedies for the treatment of T2DM. Despite a vast literature on a variety of different plant extracts that have been suggested to be beneficial in treating T2DM, there are few structured, sequential studies providing comprehensive evidence for the efficacy of a specific extract. Here, we have devised a set of screening criteria and a sequential process through which to identify likely plant-based candidates that modulate β-cell function for the treatment of T2DM (Figure 1). We have identified six major characteristics that should be present in any β-cell-directed plant-derived therapy. These characteristics include the ability to stimulate insulin secretion through activating known stimulus–secretion coupling pathways while maintaining β-cell viability, mass and insulin stores. Thus, we have plugged into this framework a number of our recent experimental studies using an aqueous GS extract, OSA^®^, to validate this screening process as a means of identifying antidiabetic extracts with β-cell-directed therapy. Although we have focused here on OSA^®^, similar screening characteristics are valid for our studies using other plant extracts such as *Costus pictus* and *Commiphora Myrrha* [62,63,64,65,66].

We show that OSA^®^ fulfilled almost all of the criteria identified for an effective antidiabetic agent through targeting β-cell function, being (i) an effective insulin-releasing agent at nontoxic concentrations; (ii) maintaining β-cell insulin content by stimulating a simultaneous increase in insulin gene transcription to avoid β-cell exhaustion; (iii) maintaining β-cell mass by protecting against apoptosis induced by cytokines in an inflammatory T2DM-like environment; and (iv) being effective at maintaining normoglycemia in vivo in a mouse model and a human cohort with T2DM after delivery by the enteral route. Investigating the portfolio of other *Gymnema* species, such as *Gymnema montanum* (GM) and *Gymnema yunnanense* (GY), extracts fulfilled some of the properties for having antidiabetic therapeutic potential through being able to (i) preserve β-cell mass via protecting against apoptosis [67] and (ii) reduce blood glucose levels, increase plasma insulin concentrations and maintain beta cell mass in animal model of diabetes in vivo [68,69,70,71,72,73]. However, more studies are required to assess the other properties of the “screening portfolio” for GM and GY.

**Figure 2:**
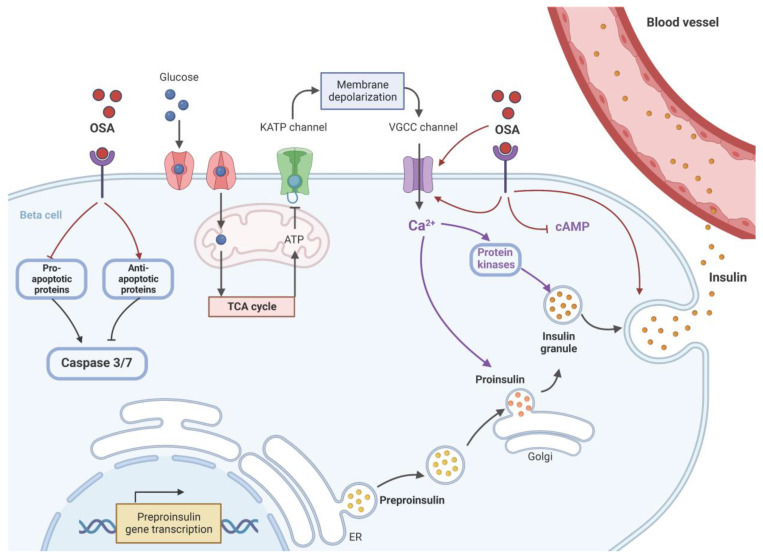
The proposed mechanism of OSA^®^ on the restoration of β-cell function. OSA^®^, either directly or through a receptor, activates voltage-gated calcium channels (VGCC) to increase intracellular Ca^2+^ ([Ca_2+_]_i_) concentrations, which, in turn, stimulate protein kinase activation. This should trigger insulin exocytosis and release. OSA^®^ may also act directly to stimulate fusion of insulin vesicles and exocytosis independently from elevations of ([Ca_2+_]_i_ and activation of protein kinases. OSA^®^ also stimulates the expression of preproinsulin to maintain a constant supply of insulin vesicles during the secretion process and to prevent the depletion of β-cells stores. To preserve β-cell mass, OSA^®^ protects against apoptosis by activating key antiapoptotic proteins while inhibiting key pro-apoptotic proteins. Both effects could reduce the levels of caspases 3 and 7 inside the β-cells. ER: endoplasmic reticulum, K_ATP_: ATP-sensitive potassium channel, TCA: tricarboxylic acid cycle (Krebs cycle). Created with BioRender.com.

The schematic diagram in Figure 2 shows proposed mechanisms through which OSA^®^, and possibly other GS extracts, improve β-cell function and viability. We suggest that future studies of plant extracts as β-cell-targeted therapeutic agents for T2DM should adopt this “portfolio” approach of identifying likely candidates to take forward to clinical trials. In addition, we propose that the outcomes of our studies using OSA^®^ have emphasized the therapeutic potential of these extracts as an inexpensive and readily available adjunctive therapy for the treatment of T2DM.

## Figures and Tables

**Table 1:** Summary of pharmacological effects of OSA^®^ and other *Gymnema Sysvestre* extracts on β-cells function in vitro and in vivo.

Name of Extract	Compound Information	Model	Described Effects and Mechanisms	Fulfilment of Screening Criteria	References
In vitro					
Alcoholic extract of GS (GS4, F2, F43)	Chemical composition of GS4 and F2 is unknown F43 may contain gymnemic acids VIII 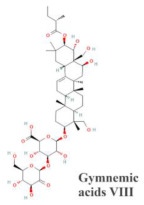	MIN6	Concentration-dependent (0.125–0.25 mg/mL) stimulation of insulin secretion High doses have deleterious effects on MIN6 cell viability by trypan blue exclusion method	1 2	[18]
		RINm5F	Concentration-dependent (0.125–0.5 mg/mL) stimulation of insulin secretion	1	
		HIT-T15	Concentration-dependent (0.125–0.5 mg/mL) stimulation of insulin secretion Increases in insulin levels is partially dependent of VGCC	1 4	
		Isolated rat islets	Stimulation of insulin secretion (0.2 mg/mL) at 2, 10 and 20 mM glucose concentrations High doses have deleterious effects on islets viability by trypan blue exclusion method	1 2	
Ethanolic extract of GS	Contains mixture of gymnemagenin and gymnemic acids I, IV and VII 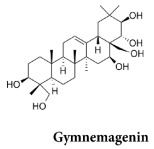 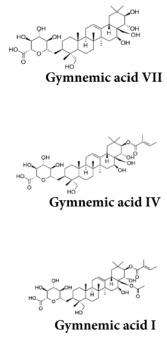	MIN6 cells	stimulation of insulin secretion (50 & 100 μg/mL) at 3, 8, 15 & 25 mM glucose Doses of 20 to 650 μg/mL have no deleterious effects on MIN6 cell viability by MTT test Increases in MIN6 cells GLUT-2 levels Reduction in the formation of ROS in H_2_O_2_-treated cells	1 2 4 5	[19]
OSA^®^	Commercially available Lyophilized powder containing molecules > 3000 Dalton in size Chemical composition of OSA^®^ is unknown May contain complex triterpenes and gurmarin	MIN6 cells	Concentration-dependent stimulation (0.06–2 mg/mL) of insulin secretion at 2 and 20 mM glucose concentrations during static insulin experiments Low concentrations of OSA^®^ (0.06–0.25 mg/mL) have no deleterious effects on MIN6 cell viability by trypan blue exclusion method Concentration-dependent increases in β-cell Ca^2+^ levels by calcium microfluorimetry Stimulation of insulin secretion is dependent of VGCC	1 2 4 4	[17]
		Isolated human islets	Stimulation of insulin secretion (0.125 mg/mL) at 2 and 20 mM glucose concentrations during perifusion insulin experiments Low concentrations of OSA^®^ (0.125–0.25 mg/mL) have no deleterious effects on human islets viability by trypan blue exclusion method Increases in insulin levels is dependent of VGCC	1 2 4	
OSA^®^	Commercially available Lyophilized powder containing molecules > 3000 Dalton in size Chemical composition of OSA^®^ is unknown May contain complex triterpenes and gurmarin	Isolated human islets	Stimulation of insulin secretion (0.125 mg/mL) at 2 and 20 mM glucose concentrations during perifusion insulin experiments	1	[16]
OSA^®^	Commercially available Lyophilized powder containing molecules > 3000 Dalton in size Chemical composition of OSA^®^ is unknown May contain complex triterpenes and gurmarin	Isolated mouse islets	Stimulation of insulin secretion (0.25 mg/mL) at 2 and 20 mM glucose concentrations during perifusion insulin experiments Elevations in preproinsulin expression & maintenance of β-cell insulin store	1 3	[15]
OSA^®^	Commercially available Lyophilized powder containing molecules > 3000 Dalton in size Chemical composition of OSA^®^ is unknown May contain complex triterpenes and gurmarin	MIN6 cells	Increases in insulin levels is partially dependent of VGCC during static insulin experiments Stimulation of insulin secretion is independent of cAMP	4 4	[21]
		Isolated mouse islets	Increases in dispersed β-cell Ca^2+^ levels by Calcium microfluorimetry Increases in insulin levels is partially dependent of VGCC during perifusion insulin experiments Stimulation of insulin secretion is independent of β-cell depolarization during perifusion insulin experiments Stimulation of insulin secretion is partially dependent on protein kinase activation during perifusion insulin experiments Stimulation of insulin secretion is independent of PKC, CamKII and cAMP	4 4 4 4 4	
		Isolated human islets	Stimulation of insulin secretion is partially dependent on protein kinase activation Stimulation of insulin secretion is independent of PKC and CamKII during perifusion insulin experiments	4 4	
OSA^®^	Commercially available Lyophilized powder containing molecules > 3000 Dalton in size Chemical composition of OSA^®^ is unknown May contain complex triterpenes and gurmarin	MIN6 cells	Low concentrations of OSA^®^ (0.06–0.25 mg/mL) have no deleterious effects on MIN6 cell viability by ATP viability test Reduction in caspase 3/7 levels in cytokines-treated cells	2 5	[20]
		Isolated mouse islets	Low concentrations of OSA^®^ (0.06–0.125 mg/mL) have no deleterious effects on MIN6 cell viability by trypan blue exclusion method and ATP viability test Reduction in caspase 3/7 levels in cytokines-treated islets Reduction in caspase 3 mRNA expression in cytokines-treated islets Increased the expression of anti-apoptotic genes and decreased the expression of pro-apoptotic genes	2 5 5 5	
**In vivo** **Animal studies**					
Crude aqueous extract of GS (5 mL/Kg)	Chemical composition of the extract is unknown	Albino rats	Improvement of glucose intolerance following GTT	6	[28]
Crude aqueous extract of GS (0.2–0.8 g/2 mL)	Chemical composition of the extract is unknown	Alloxan rats	Reduction in blood glucose levels in moderately diabetic rats Reduction in mortality rate	6	[38]
Crude aqueous extract of GS (600 mg/Kg)	Chemical composition of the extract is unknown May contain 23% of gymnemic acids	GC-induced diabetes in mice	Reduction in Fasting serum glucose	6	[26]
Crude aqueous extract of GS (2 mL/Kg)	Chemical composition of the extract is unknown	Alloxan Wistar rats	Reduction in fasting serum glucose concentrations	6	[33]
Aqueous extract of GS (GS3 & GS4) (20 mg/day)	Chemical composition of the extract is unknown	STZ rats	Reduction in fasting blood glucose levels Increases in plasma insulin levels Increases in β-cells and islet number	6	[34]
Aqueous extract of GS (GS4) (1 g/Kg)	Chemical composition of the extract is unknown	STZ rats	Reduction in serum glucose concentrations after acute and chronic treatment No change in immunoreactive insulin (IRI) response in the pancreas	6	[32]
Dihydroxy gymnemic triacetate (20 mg/Kg)	Acetone extract of GS leaves 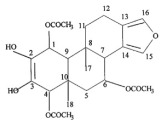	STZ Wistar rats	Reduction in Fasting plasma glucose and glycosylated hemoglobin Increases in plasma insulin levels Improvement of lipid profile	6	[24]
Dried leaves powder of GS (250 mg/day)	Chemical composition of the extract is unknown	Alloxan rabbits	Reduction in Fasting blood glucose levels Improvement of lipid profile Improvement of glucose intolerance and raised insulin level following GTT	6	[37]
Ethanolic extract of GS (100 & 200 mg/Kg)	Chemical composition of the extract is unknown	High carbohydrate fed rats	Reduction in glucose rise following adrenaline injection	6	[27]
Ethanolic extract of GS (GS4) (20 mg/day)	Chemical composition of the extract is unknown	STZ albino rats	Improvement of glucose intolerance and insulin level following GTT Reduction in glycosylated hemoglobin and glycosylated plasma proteins	6	[36]
Hydro-methanolic extracts of GS (400 mg/Kg)	Chemical composition of the extract is unknown	Alloxan Wistar rat	Reduction in blood glucose levels Improvement of lipid profile	6	[30]
Gymnemic acid I-IV (40 & 80 mg/Kg)	Commercially available 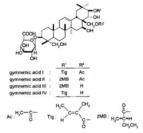	High fat diet SD rats	Reduction in Fasting blood glucose levels Improvement of glucose intolerance and insulin sensitivity Reduction in glycosylated serum proteins	6	[31]
Gymnemic acid IV (3.4–13.4 mg/kg)	Methanol extract of GS 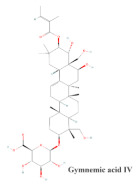	STZ mice	Reduction in blood glucose levels Increases in plasma insulin levels	6	[39]
OSA^®^ (500 mg/Kg)	Commercially available Lyophilized powder containing molecules > 3000 Dalton in size Chemical composition of OSA^®^ is unknown. May contain complex triterpenes and gurmarin	ob/ob mice	Improvement of glucose intolerance following GTT	6	[15]
**Human studies**					
Aqueous extract of GS (GS4) (400 mg/day)	Chemical composition of the extract is unknown	Human subjects with T2DM	Reduction in blood glucose, glycosylated hemoglobin and glycosylated plasma proteins Increases in serum insulin levels	6	[23]
Aqueous extract of GS (GS4) (400 mg/day)	Chemical composition of the extract is unknown	Human subjects with T1DM	Reduction in blood glucose, glycosylated hemoglobin and glycosylated plasma proteins Reduction in insulin requirements	6	[35]
Dried leaves powder of GS (10 g/day)	Chemical composition is unknown May contain oleanane and dammarene classes of triterpene saponins	Human subjects with T2DM	Reduction in fasting blood glucose levels Improvement of glucose tolerance following GTT	6	[22]
Dried leaves powder of GS (2 g/day)	Chemical composition is unknown May contain oleanane and dammarene classes of triterpene saponins	Normal human subjects	Reduction in fasting blood glucose levels	6	[37]
GS capsule (300 mg twice daily)	Commercially available Contains 25% of total gymnemic acids	Human subjects with IGT	Improvement of glucose tolerance following GTT Reduction in glycosylated hemoglobin Improvement of lipid profile	6	[25]
Dried leaves powder of GS in water (2 g three times a day)	Chemical composition is unknown May contain oleanane and dammarene classes of triterpene saponins	Human subjects with T2DM	Reduction in fasting blood glucose levels Improvement of glucose tolerance following GTT	6	[29]
OSA^®^ (1 g/day)	Commercially available Lyophilized powder containing molecules > 3000 Dalton in size Chemical composition of OSA^®^ is unknown May contain complex triterpenes and gurmarin	Human subjects with T2DM	Increased levels on insulin and C-peptide Reduction in fasting and post-prandial blood glucose	6	[16]

## Abbreviations

[Ca^2+^]_i_	Intracellular Ca^2+^
ATP	Adenosine triphosphate
CamKII	Calcium-calmodulin kinase II
cAMP	cyclic adenosine monophosphate
DAG	diacylglycerol
DPPIV	Dipeptidyl peptidase IV
EGTA	Ethylene glycol tetraacetic acid
GLP-1	Glucagon-like peptide 1
GS	*Gymnema sylvestre*
IL-1β	Interleukin-1 beta
IP3	Inositol trisphosphate
K_ATP_	ATP-sensitive potassium channel
mRNA	Messenger RNA
PI3K	Phosphatidylinositol 3-kinases
PIP3	Phosphatidylinositol (3,4,5)-trisphosphate
PIs	pseudoislets
PKCαβ	Protein kinase Cαβ
PLC	Phospholipase C
PPI	Preproinsulin
T2DM	Type 2 diabetes mellitus
VGCC	Voltage-gated calcium channel
β-cell	Beta cells
